# The Idea and Becoming of a University Across Time and Space: Ivory Tower, Factory and Network

**DOI:** 10.1007/s42438-022-00341-0

**Published:** 2022-10-05

**Authors:** Adam Matthews

**Affiliations:** School of Education and Engineering and Physical Sciences, University of Birmingham, Birmingham, UK

**Keywords:** University, Higher education, Ivory tower, Factory, Genealogy, Postdigital, Spaces, Discourse

## Abstract

The modern university has grown from small scale, elite access institution, growing out of the Enlightenment period in Europe in the early nineteenth century. Freedom to pursue knowledge and ‘dare to know’ was a key characteristic of the Enlightenment university, conceptualised here as *Mode 1 Elite Ivory Tower University*. The twentieth century saw a rapid rise in national government involvement, funding and regulating universities as a way of nation state building. This developed within social contexts of neoliberal knowledge and information economies. Market demand and regulation, seeing teaching and research as products saw huge growth of universities in size and number globally. More citizens accessed and engaged with universities as large institutions, conceptualised here as a *Mode 2 Mass Access Factory University*. Globalisation, digital technologies and a move away from big organisations and states is seeing an emergence of a *Mode 3 Universal Network University* which is universal and unbundled in complex technological and social network relationships, often underpinned by a form of capitalism which is developing neoliberal approaches aided by data collection and information networks. These three modes are developed and explored here through a postdigital lens across time (genealogically) and space (residual, dominant and emerging discourses and cultures remaining in and between institutions) as a theoretical framework with which to research and envisage the characteristics, discourse, perceptions and becoming of the current and future university.

## Introduction

The idea and purpose of a university have been debated and contested by academics, administrators, students, politicians and the wider public for centuries. Such debates centre on knowledge as a public or private good. This includes the freedom to pursue knowledge as an intrinsic good in itself, contrasting with instrumental knowledge for specific societal and individual ends. Moreover, who is involved in the becoming and being of a university is highly contested (i.e., government policy, management, students, academics, publics, private enterprise, etc.). At a structural level, universities globally have developed and grown with evermore complex relationships and involvements with the state, markets and academics (Clark 1983).

These debates have shifted as universities have grown and social and political environments have changed. Trow (1973) described the pattern of growth (and the complicated nature of it) of the modern university as one of elite access (a small and privileged number of the world accessing and forming the university), changing over time to mass higher education (many nations have targeted access rates of 50%, driving social divisions and hierarchy). Following elite and mass phases, Trow described the potential of moving to universal access and what this might mean for the nature and character of the university as an institution.

Analysis of the idea of a university is timely and important as universities grow but also taken on greater social responsibilities. Barnett (2019) describes this as a *University Challenge* with regard to division (who has access), discourse (how the many understandings of a modern contemporary university are articulated and understood by all of a population) and democracy (a public that can engage with ever growing knowledge and supercomplexity).

Recent books on the state of the university in the UK and their titles and sub-titles are prime examples of the state of flux in which the role of universities is in. The titles: *Universities Under Fire: Hostile Discourses and Integrity Deficits in Higher Education* (Jones 2022) and *Retreat or Resolution? Tackling the Crisis of Mass Higher Education* (Scott 2021) tell their own story. Moreover, in the same context there is a governmental discourse which is seeking to ‘weed out’ degrees which are deemed to be ‘low value’ in employment outcomes.

The twenty-first century has been dominated by a revolution in digital technologies which has left little or no aspects of life unaffected. Peters and Jandrić (2018) define this as the epoch of digital reason. This is not purely technical but political, social and economic. Critical challenges for the idea of a university and technological ‘disruption’ in the 2020s include digital in/exclusion, platform economics in an age of artificial intelligence, divisions of learning across humans and machines and neurotechnology, IT industry actors, datafication and unbundling of the university degree along with changing professional roles within the university (Bayne et al. 2020; Selwyn et al. 2020). In line with the epoch of digital reason, such issues and opportunities are not purely technological but are entanglements with the question of social purpose of the university and the materiality of technology (Gourlay 2021; Jones 2019; Williamson 2022).

The many possible responses to these challenges will have a direct impact upon the idea of a university and its future role in society. In this paper, I develop a framework which traces the dominant ideas of the modern university and how these discourses have developed within evolving social structures. The framework includes three modes:

- Mode 1: the university as the autonomous elite ivory tower born out of the Enlightenment period.
- Mode 2: the university as a factory producing knowledge for societal market driven needs for mass participation in the neoliberal knowledge economy.
- Mode 3: the universal networked university as a complex network assemblage of actors inside and outside of the university, with potential universal access in socially and technologically networked epoch of digital reason.

**Fig. 1:**
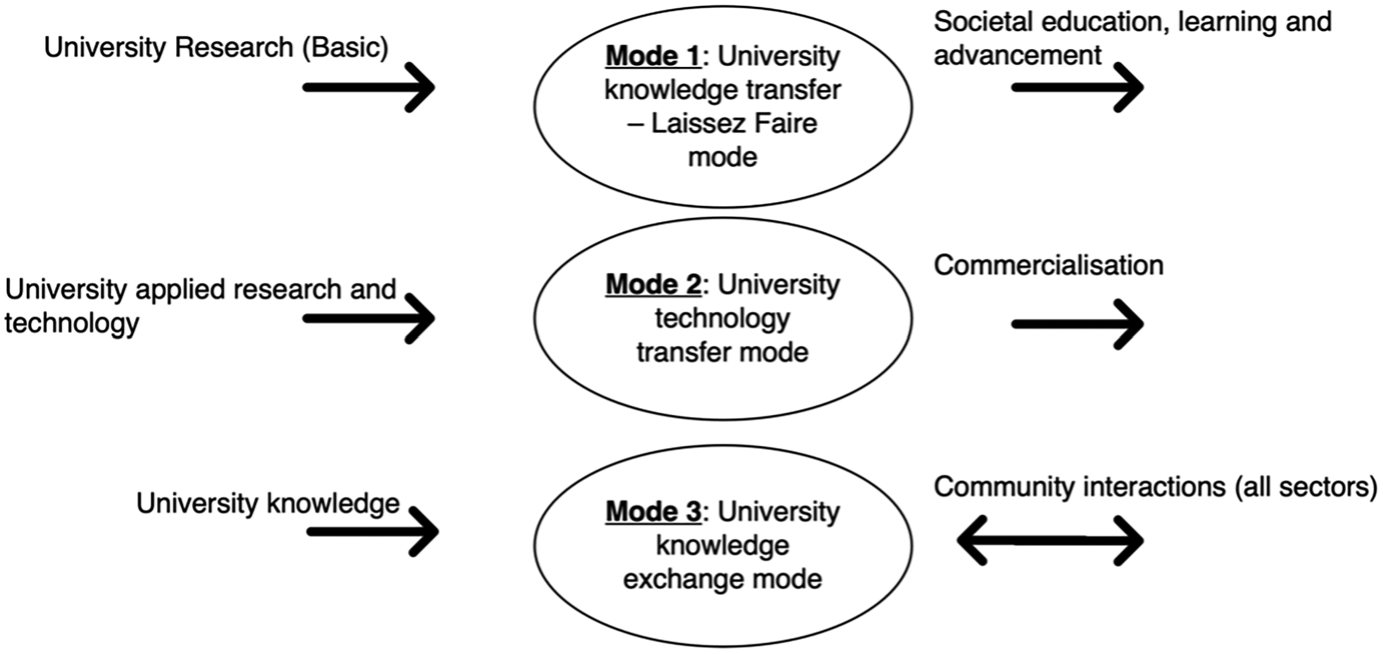
Knowledge production modes and university knowledge transfer. Based on Miller, McAdam, and McAdam (2018)

These modes (listed above and represented in Fig. 1) I argue have developed over time (genealogically) but still remain and are contested over space within and between universities.

## A Postdigital Framework Across Time and Space: Mode 1 Ivory Tower, Mode 2 Factory, and Mode 3 Network

Such developments do not emerge in neat, structured changes over time but produce sites of contestation and debate layering over each other with both a cultural memory and path dependence. Raymond Williams (2011) wrote of a *Long Revolution* which would open access to knowledge and education to develop a participatory democracy aided by new and emerging communication technologies. Similarly, Habermas saw mass communications opening a public sphere to information, ideas and discussion (Duvenage 2005). Friesen (2017) describes how media technologies are part of a *longue durée* of change in Education along with societal shifts in religion, politics and culture. Such developments are dialectical, and the purpose of the framework presented here is to conceptualise how understandings (thesis), are contested (antithesis) and become accepted discourses and practices (synthesis).

In many post-pandemic discourses, technology is the single disruptor of the university with marketing and conference soundbites (Matthews 2021). It is vitally important that technological developments are not merely seen as neutral tools to be used. Complex infrastructures and technologies do influence and change social practices in myriad ways — many of which will be unintended or unanticipated (Parvin and Pollock 2020). Jandrić and Knox (2021) describe a postdigital turn which rejects simple binaries of technological determinism as an inevitability to be kept up with and instrumentalism which sees technologies as a tool to be ‘used’. This challenges a general discourse dominated by use of technologies as end in themselves or to uncritically, with question or context enhance (Bayne 2015; Hayes 2019; Matthews 2020b, c).

The framework presented here is a higher-level postdigital convergence which is structural across time and space which takes into consideration dominant discourses, policy, and political economy (Peters et al. 2021). From a sociological perspective, such higher-level structures influence lower-level postdigital convergence such as design, curriculum, pedagogy, assessment (Matthews 2019; Fawns 2022) and knowledge production.

The purpose of this framework is to characterise and identify three modes of the university in society, how they have developed genealogically but still remain as strands of history with path dependency from the past, influencing the present and future idea of a university across spaces (Ford 2016; Lefebvre 2013) drawing upon residual, dominant and emerging cultures and discourses (Williams 1980). Such an exploration and outline is a tool to be used in empirical and conceptual work by those not only researching the university but also for those working in and leading universities. Each mode is not to be accepted or rejected in binary fashion but intended to be used to expose ideas, constatations and ultimately the nuance of the characteristics of each mode which have developed genealogically (over time) but as I hold, still remain across spaces across and between institutions.

A genealogy traces the development of an idea which shows how disruptions and changes do not begin with a blank canvas but emerge with path dependencies (Krücken 2003). Cultural memories of what has gone before play an active role in the becoming and enactment of the present and future university.

Genealogical analysis traces how contemporary practices and institutions emerged out of specific struggles, conflicts, alliances, and exercises of power, many of which are nowadays forgotten. It thereby enables the genealogist to suggest – not by means of normative argument but instead by presenting a series of troublesome associations and lineages – that institutions and practices we value and take for granted today are actually more problematic or more ‘dangerous’ than they otherwise appear. (Garland 2014: 372)

As Garland puts it, the genealogist is not only interested in how ideas have evolved and changed over time: they are interested in how these changes have been caused by relationships of power in society. Hook (2005) prescribes three principles of genealogical analysis — the genealogist must uncover (a) the role of history, (b) the nature of discourse as knowledge (social, historical and political conditions under which statements come to count as true or false) and connect that with (c) a broader analysis of the material conditions that exist socially.

Marginson (2019) argues that there have only ever been three ‘great’ ideas of the university, (1) Newman’s idea of the university as a liberal arts college in the UK, (2) Kant and Humboldt’s designs of the German research university and (3) the idea of the American research university articulated by Clark Kerr. Marginson writes:

There is much written about the University as a social form. Yet it can be argued that there are only three great ‘ideas’ of the University. One is Newman’s idea. The second, which preceded Newman in time but is more modern and more important, is the German idea developed by Immanuel Kant and Wilhelm von Humboldt. The third is the American research university idea, which was the successor to the German idea. The American idea, carried by large-scale science based institutions of social status and power; and normalised by global connections, globally visible exemplars and global rankings; is the dominant model today. (Marginson 2019: 59)

Combining Marginson’s (2019) account of the three ‘great’ ideas of the university within social contexts, I present a genealogy of how accepted discourses on the idea of the modern university has developed over time and still exist across space. I begin with the Mode 1 Elite Ivory Tower University born out of the Enlightenment including Humboldt’s bundling of teaching and research and Kant and Newman’s Enlightenment ideals. Next, the Mode 2 Mass Factory University is described by Kerr in his 1963 US *Multiversity* (Kerr 2001) within the emerging context of the neoliberal university and knowledge economy. The emerging Mode 3 Universal Network University is then analysed using the wider societal influence of the Network Society, digital technologies, globalisation and the unbundled university. The article ends with concluding thoughts and possible uses for the framework.

## Mode 1 University: The Elite Ivory Tower

Here, knowledge is universal and kept within the university walls in a self-sustaining ecosystem. The inhabitants of the ivory tower are the keepers of knowledge, and their task is to transfer knowledge from one generation to the next and from university to society. (Nørgård et al. 2019: 72)

### The Ivory Tower and the Enlightenment

As highlighted so far, each mode is embedded within a social context and epoch. The Mode 1 Elite Ivory Tower University emerged from the Enlightenment period which looked to science and philosophy to understand the world often rejecting religion as a guiding principle for societies.

ENLIGHTENMENT is man’s (sic) emergence from his self-imposed immaturity. Immaturity is the inability to use one’s understanding without guidance from another. This immaturity is self-imposed when its cause lies not in lack of understanding, but in lack of resolve and courage to use it without guidance from another. Sapere Aude! [dare to know] ‘Have courage to use your own understanding!’—that is the motto of enlightenment. (Kant 1996: 1)

The Enlightenment’s growing secularism for Kant was an opportunity for religious authority to be replaced by reason and intelligence. He connected the use of reason with freedom and describes the Enlightenment as an unshackling of human thinking. Kant’s 1798 *The Conflict of the Faculties* (1992) is one of the first pieces of writing on the structure and governance of a university. Kant describes how the university of his day was organised and how he thought that the different faculties that make up the university should work together — in healthy conflict. Kant saw this conflict between a ‘higher’ faculty (theology, law and medicine) and the ‘lower’ faculty (philosophy) (Kant 1992). In contemporary terms, we may call the higher faculty the ‘vocational’ employment-oriented disciplines, this in the late eighteenth century was the clergy, law, and medicine. By contrast the lower faculty of philosophy included disciplines as diverse as empirical natural sciences, history, geography, pure mathematics, philosophy, and the wider humanities.

Kant believed that government intervention was justified in the higher faculty to identify key employment needs. The lower faculty however should, Kant held, be free to pursue scholarship, and question, if needed, the higher faculty. The lower faculty for Kant cannot point to one ultimate truth from an authorised text or government direction. Here, we can see the complexities surrounding academic freedom and governance at an institutional and national level (this develops further in Mode 2).

Schapira (2019) puts forward the case for the contemporary university to look to the work of Kant; stating that a degree of conflict is necessary to revitalise the purpose of a university. Similarly, Evans (2008) describes the contemporary university and its place in the knowledge economy (see Mode 2) and holds that legitimate conflicts between faculties are healthy and to be encouraged.

### The Humboldtian Ideal

Twelve years after Kant’s work on the university was published, Humboldt developed the ‘German model’ in the creation of the University of Berlin in 1810 which built upon Kant’s Enlightenment ideals in institutional form. Humboldt’s vision, enacted in Berlin in 1810 was for an institution that conducted research and teaching side-by-side, not to train the employees of the future but as a place of ‘higher learning’ (Collini 2012). Linking teaching and research was an innovation in 1810 and something that is now taken as a given in many universities globally. Humboldt’s vision for what became the model of the university is a relatively short document, just 10 pages, but has had immense influence (Humboldt 1810). Humboldt’s vision of the university was as a state institution, but the role of the state was to protect academic freedoms rather than impose regulation or authority (Palfreyman and Temple 2017).

Humboldt’s principles included freedom of thought, freedom to study under any teacher, being an institution that is radically different from a school, and the treatment of students as adults with freedom and responsibility to direct their own studies (Josephson et al. 2014). Shumar and Robinson (2018) summarise the three essential functions of Humboldt’s institutional model. Firstly, the unity of teaching and research, secondly, academic freedom and thirdly philosophy (liberal arts) would all be at the core of the university.

Elton (2005) interprets Humboldt’s central idea as one of knowledge being treated as a not yet wholly solved problem. This is characterised as ‘learning in a research mode’. Humboldt’s ideal was a symbiotic link between teaching and research; research should inform teaching, but teaching should also improve research. For instance, Humboldt stated that academics making contributions to their field through research should not regard teaching as a distraction from research but the opposite in that it helps the researcher to communicate ideas and advance their field (Josephson 2014).

While influential, Humboldt’s ideals are not without critics. Dhont (2014) describes the Humboldtian tradition as being unrealistic today and the name of Humboldt is used as nostalgia and elitism. Pechar (2012) concludes that the Humboldtian ‘myth’ will decline as higher education develops and standardises further and no longer will there be an idealistic academic oligarchy presiding over the idea of a university. 

### Newman’s Idea of a University

Much writing and thinking about the purpose and aims of higher education in the UK returns to the writing of Cardinal John Henry Newman and his *The Idea of a University* (Newman 1852). In similar fashion to Kant and Humboldt, Newman subverted ideas of passing down deeply embedded unquestioned religious doctrinaire beliefs in a pre-Enlightenment university, dominated by religion (Tierney 2016). Newman wanted to introduce a new ideal, that of critical scholarship and challenging unquestioned dogma in keeping with the context of the Enlightenment period.

In the preface to *The Idea of the University*, Newman (1852) sets out his stance that a university should not be a place where the individual is trained in the skills and beliefs that have gone before them. Newman’s idea was a university for the cultivation of mind and a liberal education. This idea was not bound by disciplines. Newman believed that disciplines divided students and knowledge rather than seeing a holistic view of the world (Newman 1852). Newman was keen to point out that for him, a university was not about learning science, art, professional skill, or literature as such; rather a university education was an exercise in growth in moral and intellectual habits. Newman saw intellectual pursuits as ends in themselves and not utilitarian instrumental tools for the future.

### The Presence and Influence of Mode 1 Today

Today, many regard Newman’s vision of the university as thoroughly elitist, perpetuating the view that privileged students have the luxury of a cultivated mind and liberal education without the need to gain instrumental outcomes. Newman did however argue that by providing individuals with a broad, liberal education developing liberal, critical minds — those minds would be able to find their way in the world, finding and creating new positions and new ways of living without a focus on disciplines or professions.

A challenge for Newman and those advocating the removal of academic disciplines is grounded in impracticality. The realities of universal knowledge are starkly challenged by Fuller as ‘there’s more stuff than can be reasonably read’ (Fuller and Jandrić 2019: 200). This is in the context of the emergence of the world wide web as a social ecosystem with access to huge amounts of information. Such access has resulted in ideas of a distributed cognition and memory between human and non-human with the web as a universal medium for education (Peters and Jandrić 2018). The Mode 1 University does have its own technological influences. At the time, the printing press had led to increased public access to books, which challenged university professors’ monopolies on being the only authorities on knowledge (Clark 2008; Eisenstein 1991).

Clearly an issue with Newman’s work is his focus on the ‘gentleman’ and the consistently male gendered language throughout his discourses. Moreover, the Enlightenment period was grounded in a particular scientific rationality as well as colonial power attributed to Europe and privileged white males. As Europe colonised the world in the sixteenth century onwards, it created a global economy not only in commodities such as sugar, cotton and slaves but also knowledge. As with those who produce and consume in the material economy, the knowledge economy then and today is highly unequal with ‘celebrity’ researchers and elite institutions writing in English in leading journals (Connell 2019). Struggles and debates around the decolonisation of knowledge production, the university and classrooms, curricula and campuses are current and live (Bhambra et al. 2018) and complex (Hayes et al. 2021; Morreira et al. 2020).

A discourse of elitism of the Mode 1 Ivory Tower can also be seen in perspectives of the digital sphere. Many advocating for the ideals of the Mode 1 university also see ‘face to face’ campus based teaching as the ‘premium’ and radically at odds as to what it means to be human (Hassan 2018). Many predicted a change in perceptions and practice of digital technologies in the university following the move to online during the Covid-19 pandemic, however in many universities a narrative of ‘back to normal’ can be seen. The postdigital turn and the framework presented here allow us to go beyond surface level binaries of online/offline, analogue/digital to underlying values and philosophies of the university. Bayne et al. (2020) rise to this challenge by dissolving such dichotomies (MacKenzie et al. 2022):

Online can be the privileged mode. Distance is a positive principle, not a deficit.

Place is differently, not less, important online. Text has been troubled: many modes matter in representing academic knowledge. (Bayne et al. 2020: xi)

A dichotomy then is presented when looking at the discourse and influence of the Mode 1 University today. Notions of academic enquiry which is free and autonomous with healthy conflict (Rowland 2006) across faculties with interdisciplinary approaches is juxtaposed with an elitist and colonial power of a small group of academic elites. The colonial powers of the Enlightenment period harvested data on indigenous people and the modern version of this can be accumulated through new data technologies (Thatcher et al. 2016).

As Marginson (2019) argues, Kant, Humboldt, and Newman all have had an influence on the discourse of the present. The framework presented here sees this as genealogical over time but also arguing that many of these views remain in residual and dominant discourses. Mode 1 was not left behind but built upon as the UK and USA entered into a social and political period of neoliberal knowledge economy.

## Mode 2 University: The Mass Factory

… the university is now positioned as the producer of the future workforce through transferrable skills and professional competences. In the factory, it is no longer the university that defines, owns, and transmits knowledge to society. Performance, output, benchmarking, and societal use-value is core to the university’s mandate, and it is up to the university to substantiate that it is delivering what society demands as well as upholding a strong position in the global competition between universities. (Nørgård et al. 2019: 73)

The development of knowledge economies whereby knowledge has become a commodity has thrust the university into a position where it has grown and developed exponentially, as *the* institution which produced knowledge in the nineteenth and twentieth centuries. One might argue that the university had little choice in taking its wares to market and to produce at large factory-like scale in the social setting of a neoliberal knowledge economy. Terms such as ‘mass’ and ‘factory’ are often pejorative in relation to what can be seen by some as an elitist Mode 1 university.

Neoliberal approaches to education and other traditionally public institutions have been well documented (Stein 2008) and institutions have been overhauled under the moniker of ‘new public management’. In education, Ball (2008) defines the neoliberal epoch as characterised by terms such as ‘creative’, ‘risk-taking’, ‘innovative’, ‘entrepreneurial’ and ‘personalised’ all with a focus on consumer need. Students are now more likely to be considered consumers of the factory product and language has become embedded in institutional discourse around satisfaction and ‘student experience’ (Nixon et al. 2018; Pötschulat et al. 2021).

Specifically, in higher education, Olssen and Peters (2005) describe the changing role of the university and the academic in the neoliberal era which result in hierarchical chains of command which reduce professional autonomy or what thinkers of the university of the past have defined as academic freedom (Mode 1 University). Marketized neoliberal pressures are measured on factory like input and output, in the contemporary UK university, we can see this as the primary activities of the university, research and teaching are measured for ‘excellence’ in REF and TEF in the UK^1^ (Matthews and Kotzee 2019, 2022) with Weberian bureaucracy (Sager and Rosser 2021). Such audit culture (Power 2013) and use of metrics and rankings have been described as ‘an attack’ (Bacevic 2018; Loveday 2021) and contrasts highly with freedom to pursue knowledge described in Mode 1.

Alongside and intertwined with neoliberalism is the emergence of a knowledge economy where knowledge is capital comparable to land and labour. Knowledge capitalism for Burton-Jones (1999) is vitally important for nation states and knowledge acquisition through education and knowledge production through research. Bell (1976) described the coming of a post-industrial society and the emergence of the knowledge worker:

The central person in this society is the professional, for he is equipped by education and training to provide the kinds of skills which the post-industrial society demands. Central to the post-industrial society is the fact that the sources of innovation are the codifications of theoretical knowledge. (Bell 1976: 576)

Knowledge is vital to participatory democracy and its distribution (teaching and other forms) is as important as other resources when it plays such a vital role in society as both socio-economic and epistemic goods. A knowledge economy requires citizens to acquire knowledge throughout life and creatively identify and solve problems in a variety of contexts (Seltzer and Bentley 2001).

Gibbons (1994) termed two modes of knowledge emerging in the late twentieth century, Mode 1 and Mode 2 knowledge and its production. This maps directly to the Mode 1 and Mode 2 universities described in this article. Mode 1 knowledge is set within the confines of a discipline (i.e., biology, computer science, sociology, etc.). Mode 2 knowledge however is broader and looks to apply knowledge to real world issues. Mode 1 for Gibbons is what is often the traditional view of science — problems are set and solved in context of the academic discipline and the interests of that community. In Mode 1, the university is autonomous, seeking out knowledge under institutional and researchers’ own disciplinary terms (basic research). This is often characterised as having no applicable goal — knowledge as an end in itself — a dominant notion in the Mode 1 University.

The social and cultural influence as well as the economic conditions of Mode 2 has moved towards consumption rather than production, again in wider society as well as in education (Moore 2004). Universities as key knowledge producers have been positioned as key government and privately backed institutions building upon the idea of a university in Mode 1. Mode 2 is characterised by application — a problem is identified, and the research and production of knowledge moves along the factory production line within the Mode 2 Factory. For example, combating climate change and vaccinating against global pandemics. Mode 2 knowledge production transcends disciplinary boundaries in that the problem or need is not set within a discipline but identified (and often funded) externally. The university responds and knowledge is produced and ‘sold’. Moreover, this is one directional out of the university (contrasting two-way collaboration in Mode 3). Knowledge then becomes capital in the neoliberal environment and universities and students can take part in the marketplace both producing and consuming knowledge. This has resulted in an increased number of universities as well as an increased number of students graduating with a university degree moving from elite to mass participation (Trow 1973) in the Mode 2 University. The Mode 2 Factory can be seen with the increase of vocational training — doctors, teachers, etc. Connell (2019) describes this as moving from the production of knowledge which asks ‘why’ to ‘how’. While Kant in Mode 1 called for a healthy conflict of the faculties, in Mode 2 the higher faculty of the professions dominates.

Nash (2019) argues that terms such as neoliberalism and bureaucracy are oversimplified and choice, rationalisation are not wholly or inherently negative and calls for a social rather than marketized bureaucracy. Both Connell (2019) and Bacevic (2019) question the idea of swathes of managers armed with MBAs and corporate executives attacking the university from outside but ask for us to look to a wider neoliberal discourse on ways in which large organisations such as universities operate and the complex idea and becoming of a university.

### Kerr’s Multiversity

The third of the ‘great’ ideas of a university as described by Marginson (2019), Kerr’s (2001) Multiversity, builds upon ideas offered by Kant, Humboldt and Newman but casts them in a model particular to the USA — a model which has come to dominate globally (Marginson 2008). Kerr stated that the US universities of the 1960s were not copies of the European model but a new type of institution — not a university but a ‘Multiversity’. The Multiversity for Kerr does not see the university or the idea of one as a single vision of an institution but a *series of communities*.

Kerr talks of the university as a modern institution, comparing it to a corporation such as IBM. For instance, the University of California were at the time spending over $100 million on construction, employing over 40,000 people (more than IBM) and soon to have over 100,000 students. Mode 2 embodies the mid-twentieth century move to large corporate organisations and public institutions which have been in decline at the start of the twenty-first century (Sennett 2006).^2^ The modern university for Kerr was not envisioned and then created, like a Humboldt grand plan, but grows and develops organically, not with one vision but many competing ideas and priorities with academics, the state and markets all contributing (Clark 1983).

Kerr goes on to talk about the leadership of the university and the huge task for leaders in the context of these conflicts, tensions and in his own words — ‘wars’. In the context of this article, this tension is between the three modes of a university. For Kerr, the university was becoming a place of specialisation, a direct contrast to Newman’s ‘universal knowledge’ in Mode 1.

Kerr describes the problem facing the university of the 1960s as that of balance. Balance in particular between teaching and research, undergraduate and postgraduate focus, faculty and un-faculty (management and operations). Kerr foresaw the development of a knowledge economy and new technologies which would impact the idea of a university and also the blurring of the line between work and home life which translates as in his words: ‘The campus and society are undergoing a somewhat reluctant and cautious merger.’ (Kerr 2001: 86) Extension (lifelong learning, professional development, short courses, access to knowledge) education which offered learning opportunities and dissemination of new knowledge, was increasing as knowledge became more important to society and new technologies would increase the pace of change:

Television makes it possible for extension to reach into literally every home; the boundaries of the university are stretched to embrace all of society. The student becomes alumnus and the alumnus continues as student; the graduate enters the outside world and the public enters the classroom and the laboratory. Knowledge has the terrifying potential of becoming popular, opening a Pandora’s box. (Kerr 2001: 86)

### The Presence and Influence of Mode 2 Today

The presence of a Mode 2 Mass Access Factory University is still evident today with a strong formal and structural approach to the university institution managed and ran along industrial production lines in linear fashion with clear inputs and outputs. Research requires formal grants and investment to be conducted and publishers own the means of production for the craft of researchers. Teaching takes place in a much more formulaic manner to the university in Mode 1. Learning outcomes which are required to be evidenced are the first thing to be required when a new course is suggested and presented to students before study. Such outcomes are mechanical, and many argue remove creativity or the spirit of inquiry described in Mode 1. This along with standardisation across continents (i.e. Bologna process) and globally (Hadjianastasis 2017) embed the factory production line analogy further.

From a postdigital perspective, moves towards digital education and distance learning is not a new phenomenon exclusive to the Mode 3 Network University. A long and rich history remains globally with extension and extra mural classes for adult education, often on a part-time or informal basis. The field of cultural studies pioneered such approaches (Steele 2020) in the 1960s and beyond. However, such part-time higher education has been in steep decline (Matthews and Kotzee 2020) with many online platforms partnering with universities and driving commerciality rather than public good and social justice. This is part of the emergence of the Mode 3 Network.

Describing the contrast of the modes 1 and 2 is stark and in some ways clear cut. This in many respects is due a longer historical development in society and the university as an institution. The move from artisan to factory worker is well documented (i.e. Marx) and the academic role can see similarities to this change.

The Mode 3 Network University is an evolution of this. Factories all over the world have been broken up to make component parts of a product in different regions. Data surveillance has been ramped up on production lines aided my automation and digital technologies (Andrejevic 2020). Castells (2000) and van Dijk (2020) argue that networks have changed societies. McLaren argues that we are still in an era of factory-like production and they have just been digitalised (McLaren and Jandrić 2015).

Digital technologies hold great promise (Networked Learning Editorial Collective 2021; Networked Learning Editorial Collective et al. 2021) and potential but as positioned in the introduction, a postdigital turn provides us with perspectives beyond technological determinism and instrumentalism. The move from Mode 2 to Mode 3 then is complex and more contested as we live through it.

Trow (1973) described a move from elite (Mode 1) to mass (Mode 2) to universal access (Mode 3). University growth along with new technologies have the potential for universal access to knowledge. Next, I explore the idea of the Mode 3 Universal Network University as part of a Network Society within an epoch of digital reason.

## Mode 3 University: The Universal Network

Unlike in the mode 1 and mode 2 university-configurations, neither university nor society holds the power of definition in relation to what constitutes valuable knowledge, education, and academic development. Rather, both society and the individual institution need to treat the university as being ontologically and geographically open. Implying that they need to enter into conversation and collaboration and be committed to each other to create knowledge for an unknown and open future. (Nørgård et al. 2019: 75)

In the epoch of digital reason (Peters and Jandrić 2018) individuals are faced with huge swathes of information as well as the ability to create their own aided by digital technologies (Lee 2021). Access to information from a variety of sources is vitally important to a functioning and participatory democracy. Moreover, information and knowledge is key work of the university in both modes 1 and 2.

A discourse whereby technologies are seen as ‘used’ for our institutional or personal ends rather than acknowledging the agency of the technology and associated infrastructures dominates (Bossetta 2018; Flanagin et al. 2010; Matthews 2020c). Lessig (2002) described code as ‘law’ in how such technical infrastructures shape the social. At the turn of the twenty-first century, there was a movement which saw cyberspace as a utopian site for a new way of living, building on political movements from the 1960s counterculture (Turner 2008). Such utopianism has been dampened by threats to democracy (Barrett et al. 2021) and political polarisation characterised by claims of fake news rather than debate and compromise in a post-truth era (Fuller 2018; Morris 2021).

Information, argue Peters and Jandrić (2018), is not a neutral and quantitative ‘thing’ whereby more equals greater democracy. Global digital technologies afford greater access to information in society, greater opportunities to access university teaching and research should then follow. As well as ideas of universal access, Caplan (2018) and others^3^ claim that such access to information renders education systems obsolete. These are all key questions for the future university as a public institution in an epoch of digital reason.

The Mode 2 Factory University produces and disseminates knowledge in line with regulation and market need in a one way, producer/consumer relationship — a production line. In broader society, lines are being blurred between producer and consumer as users generate content, information, knowledge, and data (Eichhorn 2022; Hayes 2019; Thrift 2008). Mode 3 knowledge production takes a systems theory approach whereby there are many elements within a system that come together in self-rationalising ways as elements co-exist, forming creative knowledge environments. In this context, the university becomes more open both outwardly and inwardly (Carayannis et al. 2016). The nodes or elements in this network are many: industry, governments, academia and the wider publics (Carayannis et al. 2018; Carayannis and Campbell 2012). Liyanage and Netswera sum up the transition from Mode 1 knowledge production to Modes 2 and 3.

In other words, Mode 1 is not adequate to solve social problems. As a result, Mode 2 and Mode 3 have evolved combining scientific knowledge and social contexts. It is a reflexive knowledge production system with reverse communication. Namely, science speaks to society, and society speaks back to science. (Liyanage and Netswera 2022: 3)

The Mode 3 University then affects and is affected by actors inwardly and outwardly. Mode 2 knowledge has been characterised by university-industry-government relations as a ‘triple helix’ (Etzkowitz and Leydesdorff 2000). Mode 3 adds a fourth element of wider public (culture, media, values, technology, creative industries) to make a quadruple helix (Peris-Ortiz 2016; Miller et al. 2018). Figure 1 represents this genealogical development and framework developed in this article with Mode 1 involved in basic research for knowledge as an end in itself, Mode 2 as ‘applied’ and ‘useful’ produced and delivered to society, often in a commercial manner and Mode 3 building on each of these to see outside influence and collaboration externally from a range of sectors.

### Network Society in the Information Age

Technology and information play a key part of the knowledge economy in a post industrial society. Castells (2000) saw the evolution of the knowledge economy as *The Rise of The Network Society* and uses the network as technical, social and political connection and interaction. This for Castells has redefined not only work, education and economics but all social structures:

Networks constitute the new social morphology of our societies, and the diffusion of networking logic substantially modifies the operation and outcomes in processes of production, experience, power, and culture. While the networking form of social organization has existed in other times and spaces, the new information technology paradigm provides the material basis for its pervasive expansion throughout the entire social structure. Furthermore, I would argue that this networking logic induces a social determination of a higher level than that of the specific social interests expressed through the networks: the power of flows takes precedence over the flows of power. (Castells 2000: 500)

Networks for Castells are open, with the capacity to expand and innovate without limits and by describing the network as a morphology, Castells states that the network is becoming the very structure of society. Networks are global and the nodes in the network are diverse; Castells lists, amongst others: stock exchange markets, governments, television systems and the natural world as examples of nodes in the network society. Universities in Mode 3 can be added to the list. Castells proposes that the most powerful nodes in the network are the ‘switches’ in interconnecting networks: important switches are capital, management, information and technological know-how. Castells asks: in this meta-network of capital, who are the owners, producers, managers, and servants? He concludes that the answer is increasingly blurred and that clear identities such as producer and consumer and worker and owner are lost.

Duff (2022) compares the work of Bell and Castells as grand theorists of contemporary society in the information age with the latter building on the formers work. Duff states that Bell predicted what was going to happen while Castells documented social reality. They were both concerned with a development of societies from pre-industrial (Mode 1), industrial (Mode 2) and post-industrial (Mode 3):

It is not, they both see, a simple question of before and after; instead informatization, while transforming the economy in essential ways, coexists with increasingly marginalized elements of the old industrial structure, and indeed with pre- industrial elements such as farming and fishing. (Duff 2022: 4)

This quote articulates well the framework proposed in this article as a layering of social, cultural and economic development of a particular period, not cleanly displacing what has gone before but developing epochs of change as a trajectory.

The university in Mode 3 working under this social context has, in line with other organisations become international. Universities in Mode 1 and Mode 2 were local and national respectively, in Mode 3 they are global.

### Globalisation and Internationalisation of Higher Education

The Mode 3 Network University is global and internationalized. Globalization is part of the Mode 3 make up, defined as flows of technology, economy, knowledge, people, values, and ideas across borders (Knight and De Wit 1997). Rivzi and Lingard (2010) describe the change from nation state policy making to globalized perspectives around transnational economic activities fuelled by the broad concept of globalization underpinned by capital and finance, but also political and cultural changes reshaped by information technologies. Just as Castells was writing about the emergence of the Network Society in the early twenty-first century, and as predicted by Bell, scholars of higher education have closely traced the influence of globalization as ‘internationalization is changing the world of higher education, and globalization is changing the world of internationalization’ (Knight 2004: 5).

Internationalisation widens the network and increases the nodes and interactions of actors. Stein et al. (2016) identify social imaginaries and the many conceptualisations of internationalisation in higher education. Perspectives include for a global knowledge economy (competition and commodification of knowledge), as a global public good (production of democracy, prosperity, governance and knowledge), anti-oppressive approaches (offering critiques of capitalism and who is deemed as the public and what is good) to internationalization and relational translocalism (a more radical change is needed, ontologically and metaphysically). Knight (2004) looked at the national (human resources development, strategic alliances, commercial trade, nation building and social/cultural development) and institutional level (international branding and profile, income generation, student and staff development, strategic alliances and knowledge production) with key rationales being social/cultural, political, economic and academic. The benefits of an international outlook can be articulated around collaboration and knowledge sharing but critiques of internationalisation ask who has the power to determine the global agenda and the risk of hegemonic views of the university (Modes 1 and 2 established in the global north for example) are enacted (Mode 1) and sold to the world (Mode 2).

Both Modes 1 and 2 historically can be accused of colonising knowledge production and dissemination. In Mode 3, the structure of global communications and the World Wide Web have the potential for democratic access to the production and access of knowledge, there are however still large power imbalances mirroring a colonial past. Kwet (2019) describes a digital colonialism of the US empire over the global south which provides and controls a global architecture and digital ecosystem of software, hardware and network connectivity in a system of global surveillance capitalism and imperial control, setting the foundation for technological and cultural hegemony. Big Tech firms such as Facebook can set the infrastructure and governance for global communication and media (York 2021).

### Platforms and Technology

In the context of the Mode 3 Network University which is global and open to inward and outward influence, the information age and epoch of digital reason add a new layer to the idea of higher education and research. For example, public platforms such as LinkedIn provide students and academics with services and learning materials in exchange for personal data which is often sold back to the university (Komljenovic 2019). Social media such as Facebook, YouTube, etc. are part of the knowledge network which students access in social and academic life. Academics and universities are active on such platforms and actively encouraged to present their work here to show impact and communicate to wider audiences.

Habermas was optimistic that mass communication would open up and reconstruct a bourgeois public sphere (Mode 1) for all (Habermas 2011). He also saw how rational discourse espoused by Enlightenment thinkers (Mode 1) being replaced by consumption of knowledge in broadcast mass media (Mode 2) and identified that participation was required rather than consumption (Mode 3) (Duvenage 2005). The Mode 3 Universal Network opens up opportunities for such a participatory democracy and public sphere aided by universities and digital technologies. Vattimo (1992) described the transparent society in this context of increased media access not as producing an Enlightened society but a diversity of viewpoints leading to complexity. Barnett (2000) documents the coming of supercomplexity in the twenty-first century university, which I have conceptualised as mode 3 with the complexity of outside influence and collaboration, not just in two-way interaction with the university but the range of sectors and individuals involved. Those interacting in collaboration are corporate business but there is also the potential to be communities and individuals co-producing knowledge in partnership with the university for social good. Enlightenment thinkers in mode 1 may well have embraced such access to consumption and production of knowledge. However, such complexity has been often reduced to digital reason and binary truth or falsification which some argue has led to political populism and the rise of claims of fake news in a post-truth era (Andrejevic 2020; Fuller 2018).

A danger here could be that such grand theories slip into technological determinism or instrumentalism, which as described in the introduction is the antithesis of the postdigital turn. Both Bell and Castells asserted that technology interacts with economic and cultural forces in complex ways and both describe social relations being produced and shaped by technologies. Feenberg’s (Feenberg 2017; Matthews 2020a) *Technosystem* similarly incorporates markets, administrations and technologies into a coherent social theory. Castells describes the geographical development of network technologies in the specific location of Silicon Valley on the West Coast of America. The Mode 1 University was born out if the Enlightenment period in Europe, followed by the Global North’s post-industrial knowledge economy in Mode 2. Mode 3’s development and home can be found in a region of North California.

Barbrook and Cameron (1996) articulated an ideology which has developed in the region out of Cold War technology research and a military-industrial complex followed by hardware, software and now platforms. Barbrook and Cameron’s ‘Californian Ideology’ is the catalyst of bringing together the neoliberal knowledge economy and Network Society as a harmonious collaboration between ‘hippies and yuppies’, advocating for free markets and individual entrepreneurship.

The Californian Ideology... simultaneously reflects the disciplines of market economics and the freedoms of hippie artisanship. This bizarre hybrid is only made possible through a nearly universal belief in technological determinism. Ever since the 1960s, liberals - in the social sense of the word - have hoped that the new information technologies would realise their ideals. Responding to the challenge of the New Left, the New Right has resurrected an older form of liberalism: economic liberalism. (Barbrook and Cameron 1996: 5)

Such artisanal creative and autonomous ideals align with the Mode 1 University while the neoliberal and free market approach rings true with the Mode 2 Factory.

The nature of online technologies is that of global capitalism encouraging a market for products and services. This has been termed *Platform Capitalism* by Srnicek and De Sutter (2017). Such platforms have developed a gig economy with little job security fuelled by what has been described so far in this section as a coming together of technology and economics as a global struggle (Woodcock 2021).

‘Big Tech’ is thus being reproduced in Big EdTech (Williamson 2022). Large private organisations such as Amazon are influencing and governing education through infrastructural dominance (Williamson et al. 2022). DiSalvo (2022) argues that such grand narratives of design from government, industry or academia as well as technological infrastructure can lead to a cultural imperialism that ignores or suppresses creativity and resourcefulness at a local level. Peters and Jandrić (2018) caution against an ‘Internet Universality’ when the connectivity of the world offers so much potential for social, economic and cultural benefits beyond one hegemonic world view set by global elites (universities and others).

Above I made comparison with a Marxist perspective on the Mode 1 academic as an artisanal craft worker and the Mode 2 academic as a factory worker, employed by the capitalist class in the factory using the universities means of production to produce teaching and research. For Wark (2019) in wider society, a capitalist class remains, but a new powerful, ruling vectoralist class has emerged which does not rely on land or industry for power but on information and the ownership and control of platform infrastructures. The vectoralists subordinate class Wark calls ‘the hacker class’. Hackers must constantly produce new information (directly through digital work or indirectly by collecting data on our behaviour) relying heavily on intellectual property. Thus, the vecotoralist class own the information produced by the hacker class (Wark 2004). Bratton (2015) sees Castells’ Network Society as a global private infrastructure which has evolved rather than being planned which is driven by software and data. Taking these views, we might then tentatively look at the Mode 1 academic as pre-industrial artisanal craft worker, Mode 2 as industrial knowledge producer and disseminator and Mode 3 as information producer as part of the hacker class.

The epoch of digital reason, building upon Mode 1 and Mode 2 in Mode 3 is producing the ‘university on speed’ with the embedding of the Global North’s Reagan-Thatcher period of neoliberalism and Tayloristic linear production lines (Mode 2). This has been fuelled further by digital technologies and cybernetic, fast, cognitive and algorithmic capitalism with the principles of efficiency, calculability, predictability and control aided by technology in Mode 3 (Peters and Jandrić 2018).

These many versions and theories of capitalism are in constant flux and new and developing technologies are at the heart of them. Many theorists and futurists are not envisioning progressive alternatives but a roll-back to feudalism with various versions and theories (techno, digital, information, smart, etc.) linked to Silicon Valley technologies and rhetoric. Morozov (2022) asks what a platform like Uber actually is — an intermediary? A rent-taking platform between drivers and passengers? Or a transport service? Moreover, are Google and Amazon capitalists involved in production and exchange of commodities or rentiers extracting capital through assetization? These questions can be applied to the complex network of actors in the Mode 3 university.

Komljenovic (2021) provides a theoretical underpinning of rentiership in education. Digital platform owners control access, conditions and data extraction of the platforms used by universities and other educational institutions and legal and financial regulation allow for rent to be accumulated through money (paid by institutions) and data (institutional and individual users). Importantly for the university in Mode 3 is that these platforms have become embedded not just as technical infrastructure but influence policies, governance and legal issues. With such embeddedness in the network institutional policies can be influenced or by-passed altogether. Morozov (2022) questions the intellectual robustness of using comparisons with feudalism but acknowledges that rent and dispossession of money and data through legal means has parallels with land ownership and ‘accumulation by dispossession’. However, technology companies do invest in employees, physical network infrastructure and extensive research and development. Moreover, universities in some form or other are regulated by nation states even in the Mode 3 global network which further challenges emerging notions of techno-feudalism.

Such complexity goes beyond a public/private dichotomy. Universities are actively partnering with employers and private providers in a variety of contexts in line with the inward and outward Mode 3 relationship with wider society. Morris et al. (2020) describe the pressure on universities to grow ‘market share’ globally which has resulted in commercial EdTech partners (who undertake many tasks including content development, teaching, marketing and recruitment) which often result in tensions such as growing income, an identifiable market linked to current employment statistics, pedagogical approaches and efficiencies.

Universities will need to guard against this disaggregation of education, and its unintended consequences, whilst remaining relevant and active in this space, which will continue to attract interest from a wide range of private providers, including employers and new training providers. (Morris et al. 2020: 15)

Perrotta (2018) conducted an analysis of websites including universities, Online Programme Managers (OPMs), Massive Open Online (MOOC) platforms and sector commentators showing that complex commercial relationships between universities and (MOOC) platform providers and OPMs. There are now over 2500 university and private company partnerships focused on competitive recruitment and programme development (Holon IQ 2022). Perrotta concluded that the market making of such complexity is unbundling the university degree. But rather than offering flexible, online opportunities for all, such developments are stratifying along socio-economic lines. Matthews and Kotzee (2020) identified how UK universities discourse on part-time and flexible access to the university was muted (especially in ‘elite’ institutions) while digital platforms partnering with universities took up positive claims around access and flexibility. However, the latter discourse is aimed at those who can afford the career focussed high value postgraduate courses. Those who cannot afford such access are left with the non-accredited, free courses entrenching enduring forms of inequality.

For premium, we can see this as a form of the Mode 1 Elite Ivory Tower University. Such market making and maximising of elite university ‘brands’ further builds on and compounds the stratification of universities and their students (Wakeling and Savage 2015). Here, we can link back to a global elite which has the potential to reproduce inequalities when elite institutions such as the Russell Group in the UK, Group of Eight in Australia, Ivy League in the USA, U15 group in Canada, etc. Here, we see that both economic and technological influences come into play. Further, universities, OPMs, and employers are partnering ‘to create industry-ready graduates’ (Pearson n.d.) in line with discourse of the Mode 2 Factory University. These are all examples of the outside world of the university entering and becoming part of the network.

Perhaps the biggest structural unbundling that has developed has been the unbundling of teaching and research in direct opposition to the Humboldtian ideal of teaching and research as directly linked and symbiotic in Mode 1 (Matthews and Kotzee 2022). Or those small number of global institutions with the affordance of a Mode 1 position may be the only research-teaching institutions. McCowan (2017) describes the drivers of the unbundled university as both financial and pedagogical. Financial perspectives are driven both by institutions as they become more consumer focused with efficiency savings but also financially viable for student consumers (see Mode 2 Factory University). Diverse skillsets (marketing, media production, estates, etc.) and partnership from other companies can also be seen as a positive following the rapid expansion of the university as an institution and its influence on society. For McCowan (2017) an extreme of unbundling and in the context of this article, networked in Mode 3, may see the interaction between university and society became so porous that the university as an institution does not exist anymore and each unbundled element is carried out by diverse actors and technologies.^4^ For some, such networking of the university institution is a positive of collaboration and participation drawing together the best of all three modes presented here in a large and growing university institution, for others it is the breaking down of the academic freedom and autonomy characterised by Mode 1.

## Conclusion

As described in the introduction, debates on the purpose of the university and its future are key for society and one if its key public institutions. This article provides an analytical framework of three modes of the university developed genealogically over time (genealogically Garland 2014; Hook 2005) and across space(s) (Ford 2016; Lefebvre 2013) with new and residual ideas, values and cultures remaining and becoming contested in producing ideas and becomings of the university idea and institution. Futures are often depicted in binary fashion as dystopia and utopia.

In this future all universities become proper firms, owned by investors and managers. All operations work is outsourced, and all of the teachers are sessional. Staff are appointed by managers. Curricula are trimmed back to fee-earning vocational programmes. Teaching is done online by the cheapest labour available in global markets, under automated surveillance. The most profitable universities have no campuses at all, just brands, managers, and online systems. National hierarchies of university-firms exist, under a global hierarchy of English-language universities. Only those at the top conduct research. All the research they do depends on military or corporate funding. (Connell 2019: 168)

Connell sets out her own version of dystopia and, in contrast provides criteria for a good university as an institution which is democratic, engaged, truthful, creative and sustainable in which universities are interested in the critical ‘whys’ of society as well as the ‘how to’ in research and teaching (Connell 2019).

Striking a balance between utopia, dystopia, nostalgia and futures thinking is succinctly defined by Peters and Jandrić (2018):

The university as a public knowledge institution needs to reinvent a language and to initiate a new discourse that re-examines the notions of ‘public’ and ‘institution’ in a digital global economy characterised by increasing intercultural and international interconnectedness. This discourse needs to be based on an understanding of the historical and material conditions of its own future possibilities including threats of the monopolisation of knowledge and privatisation of higher education. This discourse also needs to include the prospects and promise of forms of openness (open source, open access, open education, open science, open management) that promote the organisation of creative (or expressive) labour and the democratisation of access and knowledge in the age of cognitive capitalism. (Peters and Jandrić 2018: 254)

The framework proposed in this article responds to this call for re-examination of language to analyse the present and future university idea and its futures drawing upon sociology and philosophy of the university, networked learning and theories of knowledge production (i.e. Carayannis et al. 2018; Gibbons, 1994; Nørgård et al. 2019; Trow 1973).

The Mode 1 Ivory Tower University of the Enlightenment is free and autonomous, small scale and elite. Elites hold power and global colonisation of knowledge is the outcome of such power. The research agenda is autonomous and closely linked to teaching. Open-ended enquiry is encouraged for students and academics, often in collaboration with the end goal unknown. Knowledge is an end in itself.

The Mode 2 Factory University of the neoliberal knowledge economy is highly structured along a linear production line with inputs and outputs with mass access beyond those who could work and study in the Mode 1 University. Inputs and outputs are set by nation states with policy and regulation as well as internal policy and missions. This is often set by a ‘managerial class’ alongside academics. Outcomes are identified and achieved in teaching and research.

The Mode 3 Universal Network University is the most contested as it continues to develop in a social context of information and knowledge in abundance aided but often controlled by digital platforms within, outside and crossing campus boundaries of the university institution. Universities in Mode 3 are global with opportunities for collaboration and knowledge exchange but also to dominate and colonise. Universal access to knowledge (in a variety of formats, not just formal degrees) is a key role for universities in an epoch of digital reason. In Mode 3 universities are giving over control to some and possibly all of their functions holistically as many aspects are unbundled in a variety of contexts.

Williams (1997) developed a framework of culture always being in a state of change and flux between dominant, residual, and emergent cultures and that education institutions play a key role in the transmission of the dominant culture in society. Connell (2019) reminds us that universities are places of labour — research, teaching, learning, management, administration and operations where cultures develop and are negotiated. Bryson (2008) advocates Williams’ analytical framework to be of use to all organisational analysis to understand dominant cultures but also to identify residual and emerging ideas and how the merge and form.

The purpose of this article and its conceptual framework is to layout the dominant ideas of the university over time and space as tools to be used by researchers to further develop as well as to use to analyse the idea and becoming of universities and knowledge systems. Moreover, the three modes are designed to illuminate and encourage debate on the purpose and becoming of the university as a social institution.

The university as an institution in Mode 1 as Elite Ivory Tower emerging from the nineteenth century Enlightenment period has grown and benefited from being part of a knowledge economy and has grown exponentially forming the Mode 2 University. The Mode 2 Mass Factory University grew significantly in part due to the social shift to a neoliberal knowledge economy of the twentieth century alongside greater state interest, support and regulation. Universities as the producers of knowledge grew thanks to knowledge consumption by students, industry and government following industrial revolutions. The Mode 3 Network University has seen the advance of network technologies alongside a knowledge economy expanding the university institution further. Such networked technologies and global reach offer opportunities for wider access with the potential of universal access (Trow 1973).

Much discourse of the present and future idea of a university is dominated by technology. This often ignores social and political disruptions such as the Enlightenment in Mode 1 and the neoliberal knowledge economy in Mode 2. The Mode 3 university is an expanding network of actors which are both human and non-human and growing and developing and building from the genesis of the Mode 1 Ivory Tower and Mode 2 Factory. The Mode 3 University requires analysis of complex ecologies of actors and discourses (Barnett 2018; Ellis and Goodyear 2019) in a network society and epoch of digital reason.

A universal public engagement with the Mode 3 university in and part of society provides the opportunity to be a key institution with regard to social cohesion rather than mere producer and disseminator of knowledge. A universal access, in diverse ways, to the Mode 3 University can aim to engage active citizens from all communities in more democratic two-way engagement as part of participatory democracies through creative approaches to access and co-production in both teaching and research.

Futures are plural (Urry 2016) as are the rhizomatic ruptures traced by a genealogical approach. There have been and continue to be ruptures and new directions which have and will continue to reshape and reconfigure the idea and practice of a university — this will continue but with an increased number of actors in the Mode 3 Networked University. Such expansion and opening of borders between the university, technology, society and corporate business with unbundling of the degree and academic roles and other business-orientated models could lose the identity of the university all together and the university as we know it (McCowan 2017). This must be a key concern for universities and their role in society.

Kerr stated that the university is so many things to so many different people that it must constantly be at war with itself. Kant encouraged a healthy debate and conflict between the higher and lower faculties. Humboldt bundled teaching and research to create and share knowledge. Newman advocated education in inter and trans disciplinary ways to go out and influence the world as citizens.

Fuller (2016) calls for universities to have an Academic Caesar who is a champion of the Humboldtian university but to pragmatically see through the eyes of the neoliberal university administrator to be able to lead and communicate pragmatic, desirable, feasible and viable visions of the university of the present and future within the boundaries and parameters of social and political contexts. Kenway et al. (2015) warn against universities becoming paranoid and defensive institutions responding to political pressures but to draw upon resources of hope.

The genealogical and spatial framework laid out here provides opportunities to reflect on the development of the modern university as an analytical framework across time and space but also to provoke thinking on the role of the university as a public institution now and in the future.

## Footnotes

1 On an approximately four-year cycle, universities in the UK are required to evidence in excellence in their research and teaching through a government regulatory exercise. These are independent of each other. A grading is achieved based on many criteria, one being written evidence from universities themselves. This is then linked to funding and published rankings.

2 Large corporate buildings and factories which took up large parts of towns and cities offering employment and supply chain such as Detroit and the Ford Motor Company. Many of these operations have now been globally unbundled as described in Mode 3 below.

3 Headlines such as *Elon Musk said a college degree isn't required for a job at Tesla — and Apple, Google, and Netflix don't require employees to have 4-year degrees either* are common place (Akhtar 2020).

4 An idea proposed by Wang (1975) to offer important information (teaching), accreditation (assessment), coercion (deadlines and structure), and club membership (alumni and social capital) as separate products and services.
